# Synaptic function is modulated by LRRK2 and glutamate release is increased in cortical neurons of G2019S LRRK2 knock-in mice

**DOI:** 10.3389/fncel.2014.00301

**Published:** 2014-09-26

**Authors:** Dayne A. Beccano-Kelly, Naila Kuhlmann, Igor Tatarnikov, Mattia Volta, Lise N. Munsie, Patrick Chou, Li-Ping Cao, Heather Han, Lucia Tapia, Matthew J. Farrer, Austen J. Milnerwood

**Affiliations:** ^1^Centre for Applied Neurogenetics, Medical Genetics, University of British ColumbiaVancouver, BC, Canada; ^2^Djavad Mowafaghian Centre for Brain Health, Faculty of Medicine, University of British ColumbiaVancouver, BC, Canada; ^3^Graduate Program in Neuroscience, University of British ColumbiaVancouver, BC, Canada; ^4^Department of Medical Genetics, University of British ColumbiaVancouver, BC, Canada; ^5^Division of Neurology, University of British ColumbiaVancouver, BC, Canada

**Keywords:** LRRK2, LRRK2 mutation, G2019S, Parkinson disease, electrophysiology, cortical culture, transgenic mice

## Abstract

Mutations in Leucine-Rich Repeat Kinase-2 (LRRK2) result in familial Parkinson's disease and the G2019S mutation alone accounts for up to 30% in some ethnicities. Despite this, the function of LRRK2 is largely undetermined although evidence suggests roles in phosphorylation, protein interactions, autophagy and endocytosis. Emerging reports link loss of LRRK2 to altered synaptic transmission, but the effects of the G2019S mutation upon synaptic release in mammalian neurons are unknown. To assess wild type and mutant LRRK2 in established neuronal networks, we conducted immunocytochemical, electrophysiological and biochemical characterization of >3 week old cortical cultures of LRRK2 knock-out, wild-type overexpressing and G2019S knock-in mice. Synaptic release and synapse numbers were grossly normal in LRRK2 knock-out cells, but discretely reduced glutamatergic activity and reduced synaptic protein levels were observed. Conversely, synapse density was modestly but significantly increased in wild-type LRRK2 overexpressing cultures although event frequency was not. In knock-in cultures, glutamate release was markedly elevated, in the absence of any change to synapse density, indicating that physiological levels of G2019S LRRK2 elevate probability of release. Several pre-synaptic regulatory proteins shown by others to interact with LRRK2 were expressed at normal levels in knock-in cultures; however, synapsin 1 phosphorylation was significantly reduced. Thus, perturbations to the pre-synaptic release machinery and elevated synaptic transmission are early neuronal effects of LRRK2 G2019S. Furthermore, the comparison of knock-in and overexpressing cultures suggests that one copy of the G2019S mutation has a more pronounced effect than an ~3-fold increase in LRRK2 protein. Mutant-induced increases in transmission may convey additional stressors to neuronal physiology that may eventually contribute to the pathogenesis of Parkinson's disease.

## Introduction

Despite intense research efforts in the context of Parkinson's disease (PD), the basic neurophysiology of LRRK2 remains largely unknown. Progress is possibly confounded by numerous potential roles, resulting from LRRK2 being a large multi-domain protein containing ROC, COR, kinase, WD40, and leucine-rich repeats (Cookson, 2010). Roc and COR domains are characteristic of the Ras/GTPase (ROCO) signal transductase superfamily, involved in cytoskeleton reorganization and membrane traffic (Mizuno-Yamasaki et al., 2012). Evidence suggests LRRK2 kinase substrates include tau (Kawakami et al., 2012), endophilin A (Matta et al., 2012), 4E-BP (Lee et al., 2010, 2012) and LRRK2 itself; autophosphorylation regulates its GTPase and kinase activities (Webber et al., 2011). There is consensus between several neuronal culture studies regarding LRRK2-dependent neuritic regeneration phenotypes; axon/dendrite outgrowth are most often exaggerated by LRRK2 loss and retarded by mutant overexpression (MacLeod et al., 2006; Plowey et al., 2008; Parisiadou et al., 2009; Dachsel et al., 2010; Lee et al., 2010; Lin et al., 2010; Chan et al., 2011; Ramonet et al., 2011; Winner et al., 2011; Kawakami et al., 2012). However, others have found that neurite phenotypes are robust only during the first week *in vitro* (Sepulveda et al., 2013). Comparatively little LRRK2 is expressed during this period, whereas LRRK2 levels ~double between the first and second week, both *in vitro* and *in vivo* (Biskup et al., 2007; Piccoli et al., 2011), during which time glutamatergic synaptogenesis and synapse maturation occur. In light of this, we sought to investigate LRRK2 manipulations in relatively mature neuronal networks containing functional glutamatergic synapses (>18 days *in vitro*; DIV18).

Synaptic transmission and LRRK2 have been studied at the *Drosophila* neuromuscular junction where LRRK2 loss results in synaptic bouton overgrowth, and overexpression has the opposite effect (Lee et al., 2010) leading to exaggerated phosphorylation of the vesicle cycle regulator endophilin A (endoA), impaired endocytosis and a reduced capacity for repeated synaptic release (Matta et al., 2012). LRRK2 binds several synaptic vesicle cycle proteins, including adaptor proteins 1 and 2, alpha-actinin 2, the clathrin coat assembly protein AP180, synapsin 1, VAMP2, SNAP25, dynamin 1 and synaptophysin (Piccoli et al., 2011, 2014). In cultured cortical neurons, acute RNAi knock-down of LRRK2 increases glutamatergic release probability (Pr), vesicle motility and recycling (Piccoli et al., 2011); conversely decreased glutamate release is reported in LRRK2 KO mouse pups at postnatal day 15 (Parisiadou et al., 2014). The reports of knock-down and KO in mammalian cortical neurons and brain slices suggest that LRRK2 acts at the synaptic terminal altering glutamate release (Piccoli et al., 2011; Parisiadou et al., 2014); however, the fact that these reports are directly contradictory necessitates further examination. Here we aimed to investigate LRRK2 synaptic physiology in the context of loss of function and gain of function, against which to compare and contrast LRRK2 mutant effects. The data presented here are, to the best of our knowledge, the first investigation of glutamatergic transmission in primary neuronal cultures derived from LRRK2 transgenic overexpressing (OE), knock-out (KO) and knock-in (KI) mice.

We found that LRRK2 levels differentially regulate glutamatergic synapse density and activity in neuronal cultures from KO and OE mice. Furthermore, glutamate release was increased, and pre-synaptic regulatory protein chemistry was disturbed, in cortical neurons from KI mice. The data demonstrate that endogenous expression of the most common known genetic cause of PD has marked effects upon neuronal physiology. Such neuronal dysfunction, manifested as increased activity, may place an excessive demand upon the neuronal network that may eventually contribute to the pathogenesis of PD.

## Experimental methods

### Transgenic mice and culture preparation

Non-transgenic littermate (NT) C57BL/6J mice and transgenic human wild-type LRRK2 BAC overexpressing (OE) (Melrose et al., 2010), knock-out (KO) (Hinkle et al., 2012) and G2019S knock-in (KI) mice were maintained according to Canadian Council on Animal Care regulations. KI mice were generated by inserting a floxed PGK neomycin (PGK-neo) cassette between murine LRRK2 exons 41 and 42, using C57BL/6 genomic DNA template. In this process the targeting 5′ homology arm replaced two nucleotides c.6055G>A & c.6057G>C (numbered from the ATG start codon of mouse reference sequence NM_025730). Hence, the native nucleotide sequence of murine exon 41 GAC TAC GGG ATC GCA (encoding-DYGIA-) was mutated to GAC TAC AGC ATC GCA (mutated nucleotides underlined; encoding -DYSIA-) to encode the p.G2019S pathogenic substitution. Targeting, ES clone selection, blastocyst injection and breeding of ES cell chimeras was performed to obtain germline transmission by Ozgene Pty Ltd. (www.ozgene.com). The PGK-neo cassette was removed by crossing with Cre-deletor mice and the Cre transgene was removed in subsequent breeding. The constitutive KI animals have subsequently been maintained on C57BL/6J stock (>10 generations). Heterozygote (HET) KO × HET KO breeds yielded homozygous KO and NT pups, HET OE × NT yielded HET OE pups and NT littermates. HET KI × HET KI breeds yielded homozygous, heterozygous KI and NT littermates. Primary neuronal cultures were prepared from timed pregnancy dames from background strain C57BL/6 mice with the aforementioned transgenic crosses. Tails from each embryo were genotyped during the single-pup neuronal isolation, before cells were pooled by genotype and plated. Only heterozygous KI mice that faithfully reproduce the human condition were included and are herein referred to as KI. Cultures were prepared as previously described (Milnerwood et al., 2012); briefly, cortical neurons were isolated from pups at E16.5, brains were removed and placed on ice in Hank's Balanced Salt Solution (HBSS, GIBCO). 24-well plates were seeded at 115 k cells/well in 1 ml plating medium (PM, 2% B27+1/100 penicillin/streptomycin, Invitrogen; 0.5 mM α-glutamine; neurobasal medium, GIBCO). From DIV4, 10% of media was added every 3–5 days.

### Immunostaining and image analysis

For immunocytochemistry DIV21-26 cells were fixed in 4% paraformaldehyde (PFA) + 4% sucrose for 10 min then permeabilized with methanol (3 min at −20°C), and blocked (3 × 20 min washes of 10% normal goat serum, NGS, in PBS). Primary antibodies were incubated overnight with agitation at 4°C in PBST plus 2% NGS before blocking again (10% NGS+PBS) for 1 h at RT, then applying secondary antibody (in PBST + 2% NGS) for 0.5 h (incubated at RT for 1 h, then washed 3× with PBST before 1.5 h at RT with secondary antibodies (α-mouse Alexa-488, α-rabbit Alexa 568, α-guinea pig Alexa 633 and α-chicken AMCA from Molecular probes and Jackson Laboratories). Primary antibodies were α-microtubule associated protein 2 (MAP2, chicken, abcam, ab5392, 1:2500), α-post-synaptic density protein 95 (PSD-95, mouse, Thermo Scientific, MA1045, 1:1000), α-vesicular glutamate transporter 1 (VGLUT1, guinea pig, Chemicon, AB 5905, 1:4000) and α-synapsin1 (Synapsin1, rabbit, Millipore, ab1543p, 1:500). Coverslips were slide mounted with fluoromount (SouthernBiotech) and all images were acquired on an Olympus Fluoview 1000 confocal microscope as 0.4 μm z-stacks at 60× magnification (flattened with the max projection function for cluster analysis) and at 40× for cell density counts. Single 60× images (cluster analyses) or mosaics of 40× images were used for analyses. Excitation and acquisition parameters were constrained across all paired (culture) acquisitions. For co-localization analysis, stacks in all three channels contained all visible dendritic MAP2 staining and images were manually thresholded and binarised with experimenter blind, and quantification was conducted on masked dendritic image fields (MAP2 was expanded 5 pixels to catch pre-synaptic elements). Cluster colocalization was calculated as percentage of PSD95 clusters overlapping with VGluT1 clusters in Cell Profiler (analysis pipelines available on request) and data expressed as mean ± s.e.m., where n is average per image field (5–15 images per culture) from a minimum of 3 independent cultures (culture n in brackets). For neuronal densities, soma counts (by MAP2) were manually produced with experimenter blind to genotype on 5–6 image fields from a minimum of 3 independent cultures (culture n in brackets).

### Electrophysiology

Whole-cell patch-clamp recordings were performed on cortical cells at DIV21–26 in voltage clamp at Vh −70 mV and the membrane test function was used to determine intrinsic membrane properties ~1 min after obtaining whole-cell configuration, as described previously (Tapia et al., 2011; Kaufman et al., 2012; Milnerwood et al., 2012; Brigidi et al., 2014). Briefly, neurons were perfused at room temperature with extracellular solution (ECS) containing (in mM unless stated): 167 NaCl, 2.4 KCl, 1 MgCl_2_, 10 glucose, 10 HEPES, 2 CaCl_2_, pH 7.4, 300 mOsm. Tetrodotoxin (TTX, 0.2 μM), and picrotoxin (PTX, 100 μM, except when analyzing GABA currents) were added before use. Pipette resistance (Rp) was 3–6 MΩ when filled with (in mM): 130 Cs methanesulfonate, 5 CsCl, 4 NaCl, 1 MgCl_2_, 5 EGTA, 10 HEPES, 5 QX-314, 0.5 GTP, 10 Na_2_-phosphocreatine, and 5 MgATP, 0.1 spermine, pH 7.2, 290 mOsm. Data were acquired by Multiclamp 700 B amplifier and signals were filtered at 2 kHz, digitized at 10 kHz, and analyzed in Clampfit10 (Molecular Devices). Tolerance for series resistance (Rs) was <25 MΩ and uncompensated; ΔRs tolerance cut-off was <10%. mEPSCs and mIPSCs were analyzed with experimenter blind to genotype using Clampfit10 (threshold 5pA mEPSC, 10pA mIPSC); all events were checked by eye and monophasic events were used for amplitude and decay kinetics, while others were suppressed but included in frequency counts, as in Tapia et al. (2011), Kaufman et al. (2012), Milnerwood et al. (2012), Brigidi et al. (2014). Data are presented as mean ± s.e.m. where n is cells from a minimum of 3 separate cultures (culture n in brackets).

### Western blot

Cultures were scraped from individual coverslips at DIV21 in 100 μl of LDS loading buffer (Life Technologies). 15 μl of lysate was resolved using SDS-PAGE on a NuPage 4–12% Bis-Tris gel (Life technologies) and transferred onto a PVDF membrane (Millipore) at 25 V for 100 min at room temperature. Membranes were incubated overnight in 2% BSA/TBST with the following primary antibodies; α-Endophilin A (EndoA, mouse, Thermo Scientific, WH0006456M1, 1:1000), α-vesicle associated membrane protein 1 (VAMP1, rabbit, Synaptic Systems, 104 002, 1:2500), α-vesicle associated membrane protein 2 (VAMP2, rabbit, Synaptic Systems, 104 202, 1:2500), α-synaptojanin 1 (SynJ1, rabbit, Synaptic Systems, 145 003, 1:1000), α-dynamin 1 (DNM1, rabbit, Thermo Scientific, PA1-660, 1:1000), α-synapsin 1 (rabbit, Millipore, AB1543P, 1:5000), α-phophoserine 9 synapsin 1 (rabbit, Thermo Scientific, PA1-4604, 1:2500), α-phosphoserine 603 synapsin 1 (rabbit, Cell Signaling, 2311, 1:2500). Secondary antibodies (HRP conjugated α-mouse and α-rabbit, Santa Cruz Biotechnology) were used at 1:5000. BIO-RAD ChemiDoc™ MP Imaging System was used to detect the signal, which was quantified using inbuilt Image Lab software (Bio-Rad).

### Proteinsimple^©^western analysis

Protein levels were quantified using an automated capillary-based size sorting system (O'Neill et al., 2006), “*WES*” from ProteinSimple. All procedures were performed with manufacturers reagents according to the user manual. Briefly, 8 μL of cell lysate was mixed with 2 μL of 5× fluorescent master mix and heated at 95°C for 5 min. The samples, blocking reagent, wash buffer, primary antibodies, secondary antibodies, and chemiluminescent substrate were dispensed into designated wells in the manufacturer provided microplate. Following plate loading the separation and immunodetection was performed automatically using default settings. The data was analyzed with inbuilt Compass software (Proteinsimple). Primary antibodies used were α-synapsin 1 (rabbit, Millipore, AB1543P, 1:7500), α-phosphoserine 603 synapsin 1 (rabbit, Cell Signaling, 2311, 1:2500), and α-glyceraldehyde 3-phosphate dehydrogenase (GAPDH, rabbit, Cell Signaling, 2118S).

### Statistical analyses

Analyses were performed using Prism6 software (Graphpad, Inc.). Direct comparisons were made by Student's *t*-test (2- tailed, herein *t*-test) and multiple comparisons by appropriate analysis of variance (ANOVA) and post-tests as detailed in the text.

## Results

### LRRK wild type, overexpression and G2019S mutant levels in cortical neuron cultures

As LRRK2 is implicated in PD, a disease characterized by nigrostriatal dysfunction, we concluded it would be appropriate to study LRRK2's synaptic activity in cortical cells (CTX) given their input into the striatum is modulated by nigrostriatal dopamine.

**Figure 1:**
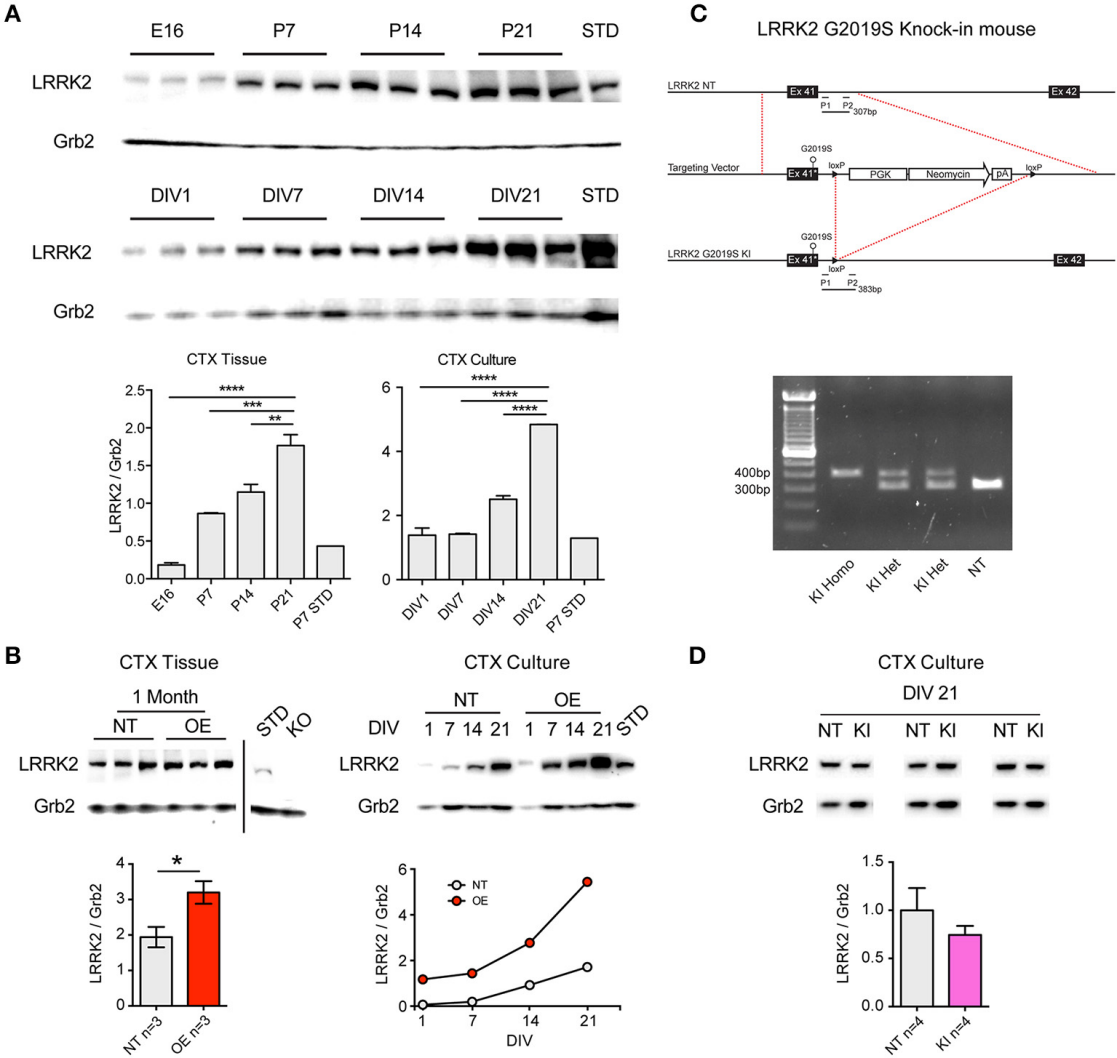
**Age-dependent LRRK2 expression *in vivo* and *in vitro* and generation of LRRK2 G2019S knock-in mice. (A)** Representative western blots showing LRRK2 expression at embryonic day 16 (E16) that increases over postnatal days 7-21 (P7-21) in mouse cortex (CTX). Positive control lysate from non-transgenic (NT) P7 CTX was used as a protein standard (STD). A similar pattern of increasing LRRK2 expression is observed in primary neuronal cultures from mouse CTX from 1 to 21 *days in vitro* (DIV). Semi-quantitative analysis expressed as LRRK2 relative to Grb2 loading control demonstrates significantly increasing LRRK2 levels over time in both CTX tissue and CTX cultures. The increase *in vivo* at P21 and *in vitro* at DIV21 occurs to a similar extent, relative to P7 STD (~5-fold). *n* = 3 Independent cultures ^**^p < 0.01, ^***^p < 0.001, ^****^p < 0.0001 by ANOVA and Bonferroni post-test. **(B)** In lysate from LRRK2 OE mouse CTX, LRRK2 protein is increased relative to NT littermates at 1 month (^*^p < 0.05 by ANOVA and Bonferroni post-test) and is absent in knock-out mouse (KO) CTX. In CTX cultures from OE mice LRRK2 expression is ~3-fold increased over NT littermates at DIV14-21. **(C)** Production of LRRK2 G2019S knock-in mouse model; Exon 41 of the endogenous murine LRRK2 (NT, top) was replaced with G2019S-containing neomycin cassette (middle), prior to cassette excision and retention of the loxP cut site (LRRK2 G2019S KI, bottom). Forward and reverse PCR primers (P1 and P2) were designed to amplify the regions flanking the loxP site, resulting in a 307bp fragment from NT endogenous LRRK2 and a 383bp fragment from each allele of G2019S KI. **(D)** PCR products reveal clear separation between the predicted band sizes and simple genotyping of NT, heterozygous (HET, herein KI) and homozygous (Homo) KI mice (left). Examples of LRRK2 western blot of lysates from three of the independent paired cultures (pooled KI and NT littermate pups) used in all subsequent experiments. There are no significant differences in the levels of LRRK2 protein in KI CTX cultures at DIV21.

Similarly to increases previously reported in whole-brain lysate (Biskup et al., 2007), western blotting for LRRK2 protein showed that levels increase rapidly during the 2nd and 3rd postnatal week in the cortex of non-transgenic (NT) mice (Figure 1A). We observed that this pattern is preserved in primary neuronal cultures of CTX cells over the first 3 weeks *in vitro* (Figure 1A), in agreement with others (Piccoli et al., 2011). As LRRK2 protein levels are relatively low over the first week, and because neurite phenotypes may be lost by the second week *in vitro* (Sepulveda et al., 2013), we decided to investigate the effects of LRRK2 manipulations upon synaptic function in neuronal networks of cortical cultures aged >21DIV. LRRK2 is absent in cortical tissue from LRRK2 knock out (KO) mice (Figure 1B) and LRRK2 levels are 2–3-fold increased in human wild-type LRRK2 overexpressing (OE) mouse cortex (*p* = 0.04) and this pattern is maintained in cortical cell cultures (Figure 1B). In order to study the effects of *LRRK2* mutations in a genetically and physiologically appropriate manner, we generated G2019S knock-in (KI) mice (Figure 1C). Founders were backcrossed onto our in-house strain >20 generations and, as predicted from similar lines (Herzig et al., 2011); our *LRRK2* KI mice are viable, healthy and breed well. Successful mutation of the endogenous mouse *LRRK2* gene, by insertion and subsequent removal of the cassette, results in a slightly longer PCR product in mutation-carrying mice, due to the residual loxP site (Figures 1C,D). LRRK2 protein levels in cortical cell cultures prepared from G2019S KI mice were comparable to NT (Figure 1D, *p* = 0.5).

### Excitatory synapse function in cortical cultures from LRRK2 KO and OE mice

We first assessed synaptic transmission in cortical cultures from KO and OE mice by electrophysiological recording and analyses of membrane properties and spontaneous activity in the form of miniature excitatory post-synaptic currents (mEPSCs) at 21DIV.

**Figure 2:**
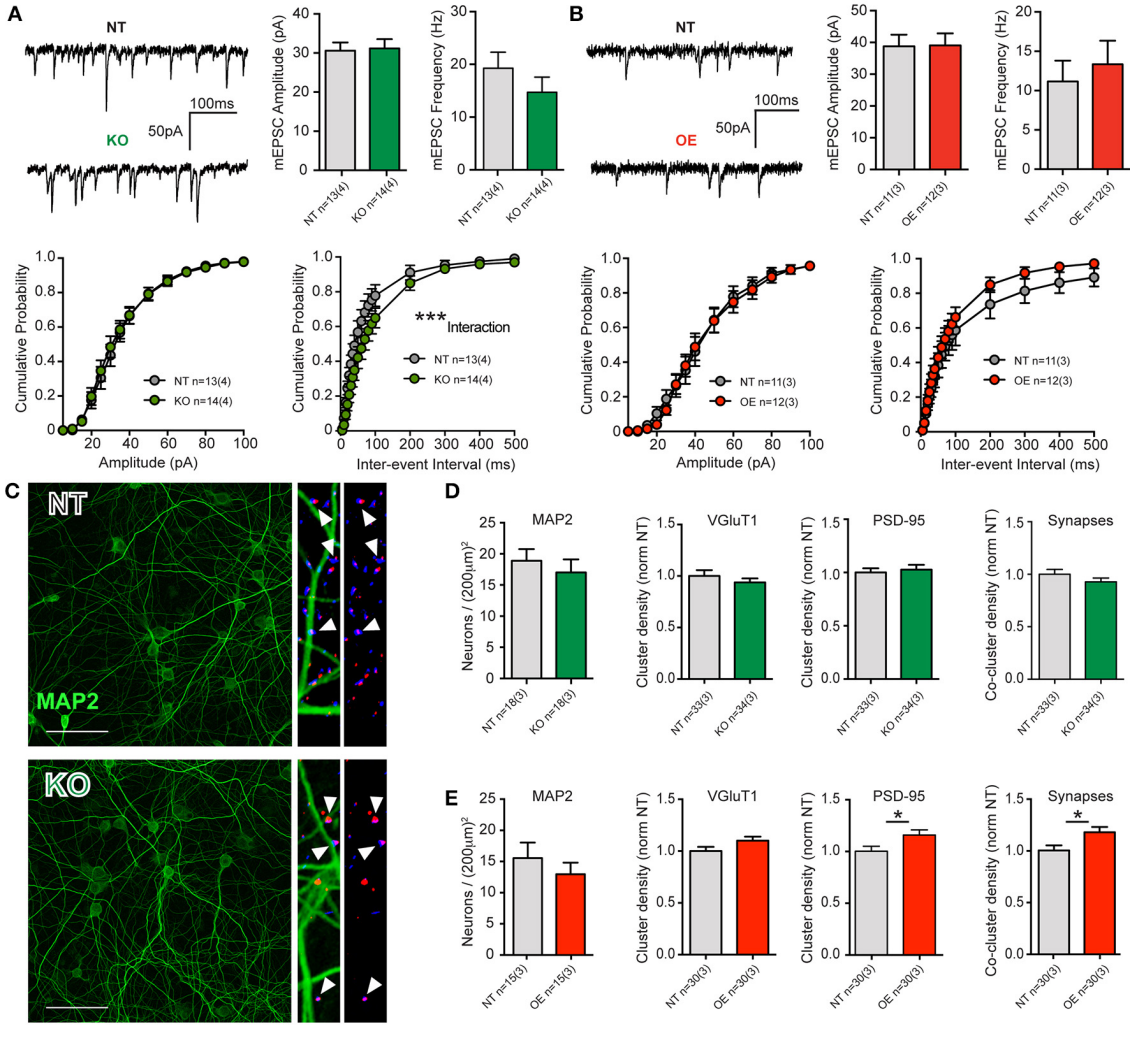
**LRRK2 levels subtly alter excitatory transmission and synaptic architecture. (A)** Whole-cell patch-clamp recordings of neurons in DIV21 CTX cultures from KO mice. (Top) Example traces of miniature excitatory post-synaptic currents (mEPSCs) mediated by glutamatergic AMPA-type glutamate receptors (AMPARs, left). Quantification of mean mEPSC amplitude and frequency shows no significant difference between genotypes (right). (Bottom) Cumulative probability analysis found no significant differences in mEPSC amplitudes, but did detect a significant interaction between inter-event intervals (IEIs) and genotype (2-way RM-ANOVA ^***^p ≤ 0.001), due to generally longer IEIs (indicative of lower frequency) in KO neurons. **(B)** Example traces of mEPSCs in DIV21 CTX cultures from OE mice (left). Quantification of mean mEPSC amplitude and frequency shows no significant difference between genotypes (right). (Bottom) Cumulative probability analysis found no significant differences in mEPSC amplitues or IEIs in OE neurons. **(C)** Cultures were stained for neuronal microtubules (MAP2, green) and excitatory pre-synaptic (VGluT1, blue) and post-synaptic (PSD-95, red) markers for neuronal density and synapse (VGluT1+PSD-95 co-clusters) measurements. Left: 40× imaging of MAP staining (bar = 100 um). Right: expanded ROI from 63× images of synaptic markers overlayed with and without MAP2. Co-clusters (white arrow heads) indicative of excitatory synapses are generally located outside of the MAP2 dendritic microtubule scaffold, upon dendritic spines that do not contain microtubules. **(D,E)** Both KO and OE neuronal densities were similar to those of their respective NT littermate cultures (by MAP2 soma counts) as were their total dendritic areas (not shown). **(D)** Although cluster intensities were significantly reduced in KO cultures (see text) and they exhibited a trend toward fewer synapses, there were no significant differences in the density or size of VGluT1 clusters, PSD-95 clusters or co-clusters. **(E)** In OE neurons, there was no significant difference in VGluT1 cluster density, despite a strong trend. There were significantly more PSD95 clusters and synaptic co-clusters in OE neurons ^*^p ≤ 0.05 by Student's *t*-test.

There were no significant differences in intrinsic cell membrane properties (capacitance, resistance and decay Tau, not shown), nor were there any differences in mean event frequencies or amplitudes in either KO or OE cortical cells, relative to NT littermate cells (Figures 2A,B). The data indicate that total membrane area and intrinsic excitability are unaltered and that quantal charge (vesicular glutamate content), the number of post-synaptic AMPA receptors and their sensitivity are equivalent to NT, regardless of the loss or overexpression of LRRK2. There were trends toward decreased and increased release frequency in KO and OE cultures, respectively; however, the only significant effect was a strong interaction between genotype and inter-event interval by cumulative probability analysis in KO cells. The data suggest that excitatory transmission is grossly normal, regardless of the absence or overabundance of LRRK2 protein.

Event frequencies are used to infer differences in synaptic probability of release (Pr) or synapse number, both of which may be altered by cell density. Neither neuronal soma counts (MAP2 stained, Figures 2C–E), nor cell viability assays (not shown), revealed any difference between KO or OE cultures, with respect to their NT controls. In order to conclude that a similar event frequency is attributable to a similar Pr, synapse density must also be determined. In cultures from KO mice, immunocytochemical staining to label pre-synaptic (vesicular glutamate transporter 1, VGluT1) and post-synaptic (post-synaptic density protein 95, PSD-95) structures showed no significant change in the mean dendritic density of either marker, or mean synapse density (estimated by VGluT1+PSD-95 co-localization). Although the size and density of VGluT1 and PSD-95 clusters was equivalent, we found that the mean signal intensity of both markers was significantly reduced in KO mice (VGluT1: NT = 0.21 ± 0.01 a.u., KO = 0.16 ± 0.01 a.u., *p* = 0.0026, MW U = 309. PSD-95: NT = 0.14 ± 0.01 a.u., KO = 0.11 ± 0.01 a.u., *p* = 0.0001, MW U = 163). Conversely, in OE cultures we observed a significant increase in the density of PSD-95 clusters, relative to NT controls, that was accompanied by a significant increase (17.6%) in synapse density (*p* = 0.017, Figures 2C–E) but no alteration to signal intensity.

Together, the data demonstrate that constitutive loss of LRRK2 does not prevent neuronal survival or synaptic network maturation, but does result in subtle negative alterations to synaptic proteins and release probability. Furthermore, the 2–3 fold overexpression of human wild-type LRRK2 had no marked effect upon neuronal survival or synaptic network maturation but did produce an increase in excitatory synapse density in 3-week-old cortical neurons.

### Increased synaptic transmission G2019S knock-in mouse cultures

The data suggest that chronic loss of LRRK2 function induces only modest negative effects upon glutamate synapses, and that LRRK2 overexpression produces an increase in synapse connectivity. This information provides the requisite foundation against which to infer gain- or loss-of function effects in PD mutants, which was the primary goal of this study. To investigate the specific effects of LRRK2 mutations we prepared cortical cultures from G2019S knock-in mice (Figures 1C,D) that carry the most common disease-linked mutation in the endogenous murine protein under appropriate expression patterns and levels (Figures 1C,D).

**Figure 3:**
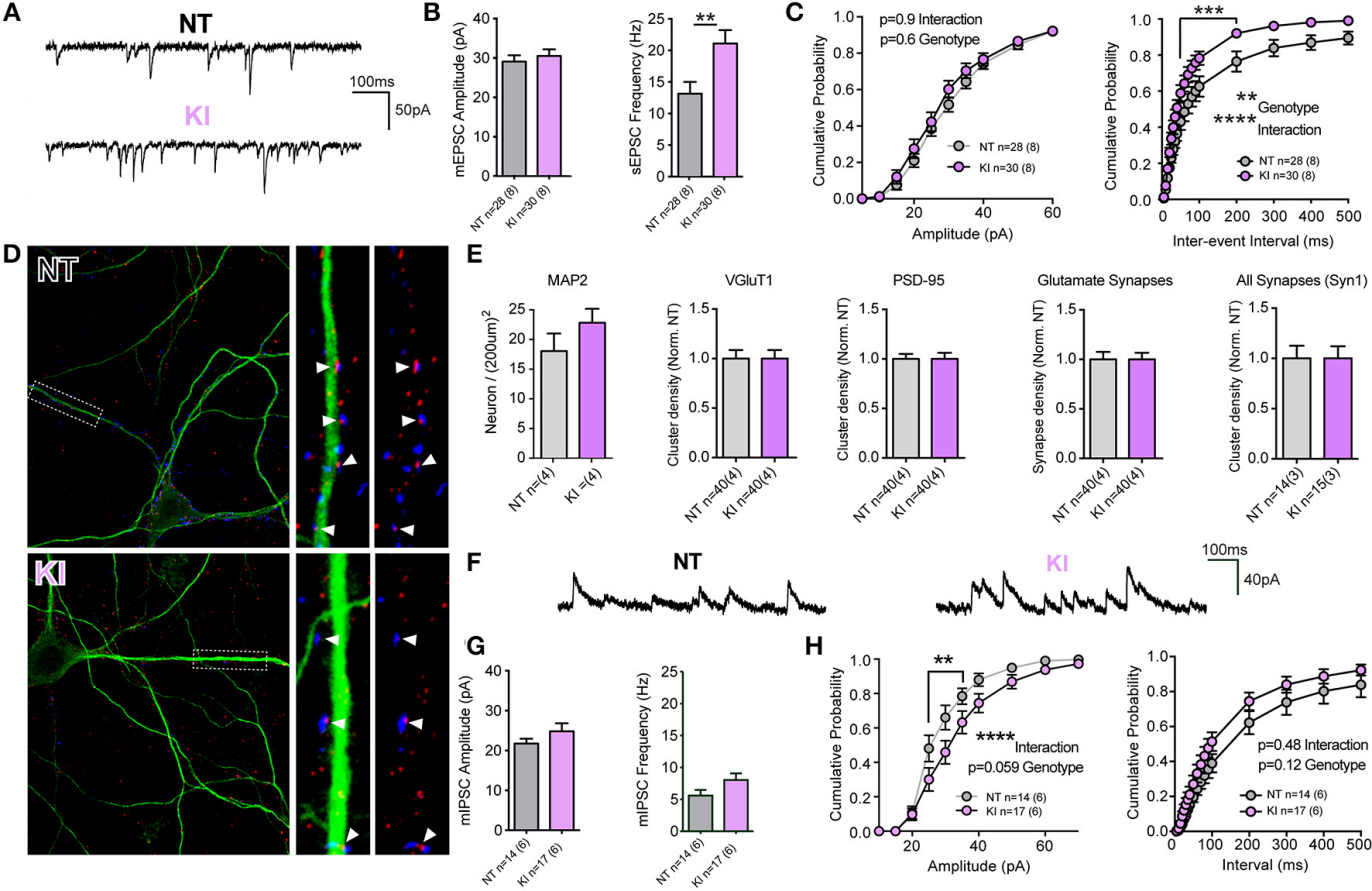
**Increased excitatory transmission and altered GABA currents in G2019S KI cortical neurons. (A-C)** Whole-cell patch-clamp recordings of neurons in DIV21 CTX cultures from KI mice. **(A)** Example traces of mEPSCs. **(B)** Quantification of mean mEPSC amplitude and frequency shows no significant difference in amplitude, but significantly higher frequency of events in KI neurons (^**^p ≤ 0.01 by Student's *t*-test). **(C)** Cumulative probability analysis found no significant differences in mEPSC amplitudes, but revealed a significant main effect of genotype and interaction between IEI and genotype (2-way RM-ANOVA, ^**^p ≤ 0.01, ^****^p ≤ 0.0001, values between 40 and 200 ms were also significant by Bonferroni post-test ^***^p ≤ 0.001), due to shorter IEIs (indicative of higher frequency) in KI neurons. **(D)** Cultures were stained (as in Figure 2) for MAP2 (green) and VGluT1 (blue) and PSD-95 (red). Left: 60× 2-times zoom of individual neuron staining. Right: expanded ROI from the 63× image showing synaptic markers overlayed with and without MAP2. Co-clusters are highlighted (white arrow heads). **(E)** KI neuronal densities were similar to those of NT littermates as were total dendritic areas (not shown), there were no differences (or trends) in the density of VGluT1 clusters, PSD-95 clusters or co-clusters (glutamate synapses). Similarly there were no differences in the density, size or intensity of synapsin 1 (Syn1) clusters, present at all glutamatergic and inhibitory synapses. **(F)** Example traces of miniature inhibitory post-synaptic currents (mIPSCs). **(G)** Quantification of mean mIPSC amplitude and frequency shows trends, but no significant differences in event amplitude or frequency of events in KI neurons. **(H)** Cumulative probability analysis revealed a highly significant interaction (and nearly significant genotype effect) due to increased mIPSC amplitudes in KI neurons (2-way RM-ANOVA, ^****^p ≤ 0.0001, values between 25 and 35pA were significant by Bonferroni post-test ^**^p ≤ 0.01). There was no significant main effect of genotype on mIPSC IEIs or interaction (despite a trend to higher frequency) in KI neurons.

Unlike KO or OE cells, the intrinsic cell membrane properties of DIV21 cortical cells from KI mice and littermate controls exhibited some modest differences; membrane resistances were not significantly different (*p* = 0.96) but membrane capacitance trended toward being increased in KI cells (Cm: NT 86.7 ± 4.9, KI 100 ± 5.0, *p* = 0.06) and membrane decay Tau was significantly slower (by non-parametric, but not by parametric Student's *t*-test. Tm: NT 1.6 ± 0.1, KI 1.9 ± 0.1, Mann Whitney *p* = 0.03). Analysis of mEPSCs demonstrated no difference in the mean amplitude of events (Figures 3A,B), but there was a significant increase (36.5%) in the mean frequency of excitatory transmission onto KI cortical cells, relative to NT littermate cells (*p* = 0.007, 21.06 ± 2.1 and 13.37 ± 1.8 Hz, respectively, Figures 3A,B). To further examine differences in mEPSCs between KI cortical cells and those from littermates, cumulative probability analysis was conducted for each cell and genotype means generated (Figure 3C). By 2-way RM-ANOVA, there was no main effect of genotype, nor was there a significant interaction between genotype and event amplitude (Figure 3C, right); however, as predicted from increased KI mean frequency, there was a highly significant main effect of genotype upon mEPSC inter-event intervals and interaction between genotype and frequency (Figure 3C, right). The results suggest excitatory transmission is significantly increased by the G2019S mutation in cortical neurons.

To determine whether increased frequency in KI culture is a result of either elevated Pr or increased synapse density, cell counts and synaptic staining was performed (Figures 3D,E). There were no significant differences in cell density, VGluT1 or PSD-95 cluster densities or excitatory synapse density in cultures from KI mice (relative to NT controls). Thus, the data demonstrate that increased excitatory synaptic event frequency in KI mice is likely due to increased Pr at a similar number of synapses.

To determine whether increases in synaptic release were specific to glutamatergic synapses, we stained cultures for the pre-synaptic protein synapsin 1 (present at both glutamatergic and GABAergic terminals) and recorded GABAergic miniature inhibitory post-synaptic currents (mIPSCs, Figures 3E–H). There were no significant differences in the number (or intensity; not shown) of synapsin 1 clusters in cultured KI neurons (Figure 3E, right), nor were there significant differences in cell mean mIPSC amplitudes and frequencies (Figures 3F,G). Cumulative probability analysis demonstrated no main genotype effect upon either mIPSC amplitudes or inter-event intervals, despite a strong trend in both (Figure 3H). There was a highly significant interaction between genotype and mIPSC amplitude. The data demonstrate that there may be subtle alterations to inhibitory synaptic transmission induced by physiological levels of the G2019S mutation, but also that excitatory synaptic release appears to be particularly sensitive to the PD associated mutation in KI mouse cortical cells.

### Decreased phosphorylation of synapsin 1

**Figure 4:**
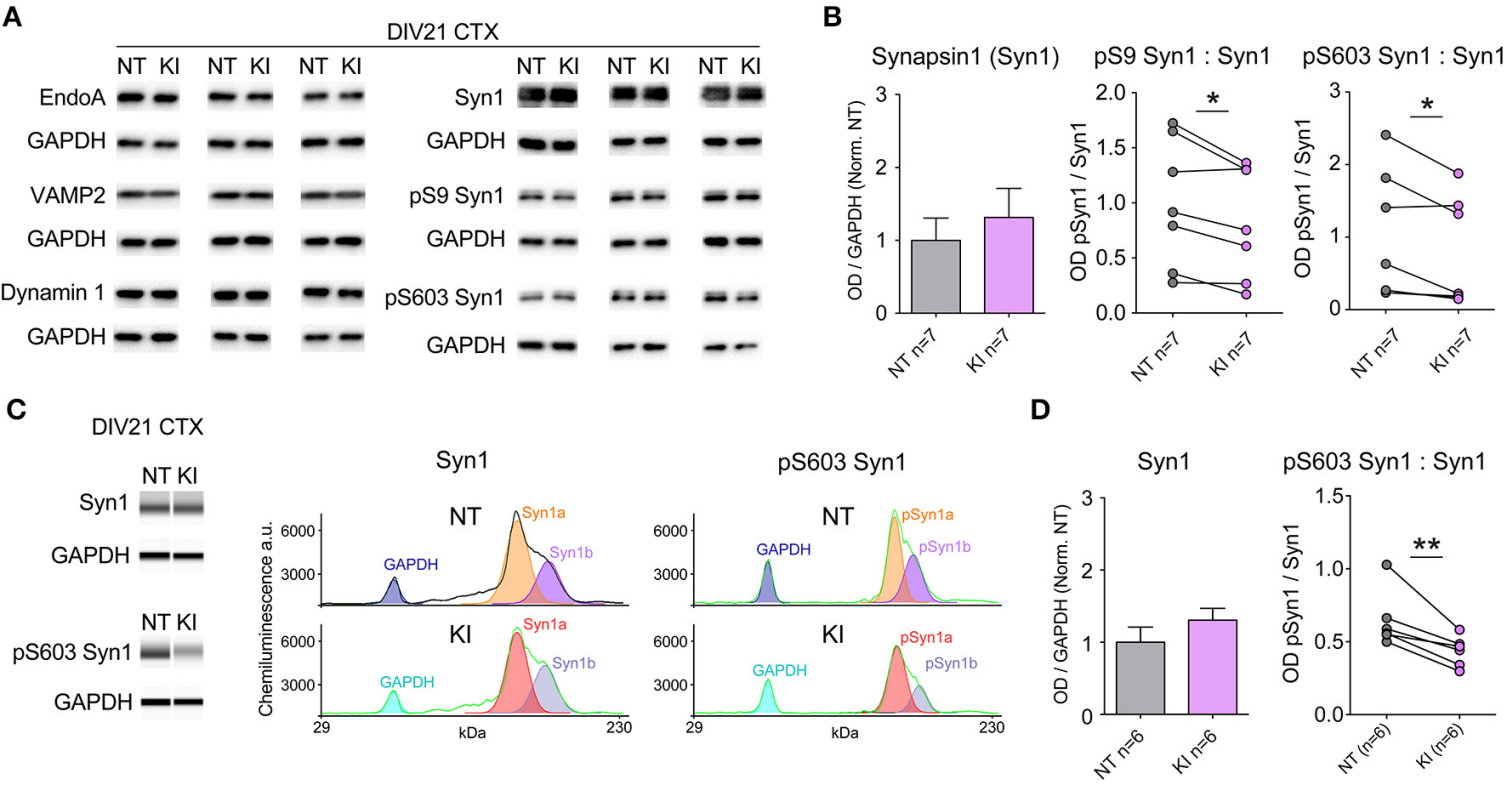
**Reduced Synapsin 1 phosphorylation in KI cortical neurons**. Levels of pre-synaptic proteins in DIV21 CTX cultures were assayed by standard western blotting and verified via *WES* automated capillary-based size sorting system. **(A)** Representative western blots of EndophilinA (EndoA), vesicle associated membrane protein 1 (VAMP1), vesicle associated membrane protein 2 (VAMP2), dynamin 1, synapsin1 (Syn1), phosphoserine 9 synapsin1 (pS9 Syn1), and phosphoserine 603 synapsin1 (pS603 Syn1). **(B)** Quantification of synapsin1 levels and associated phosphorylation sites. Synapsin1 levels were similar between NT and KI however the ratio of phosphorylated synapsin1 was significantly reduced at both sites. **(C)** Standard western blot results were verified using the WES automated capillary-based size sorting system for the S603 phosphorylation site. Representative pseudo-gels (left) and electropherograms (right) exported from the WES compass analysis software. **(D)** Quantification of synapsin1 and pS603 synapsin1 confirmed significant reductions pS603 synapsin1. Data expressed relative to GAPDH and normalized to NT, ^*^p ≤ 0.05 ^**^p ≤ 0.01 by paired Student's *t*-test.

Evidence shows LRRK2 binds several pre-synaptic release regulatory proteins including synapsin 1, VAMP2, dynamin 1 and Endo A (Piccoli et al., 2011, 2014; Cirnaru et al., 2014; Stafa et al., 2014) and LRRK2 kinase activity regulates the phosphorylation state of EndoA that is required for efficient endocytic vesicle formation and maintenance of repeated release events (Matta et al., 2012). We probed the same cortical neuronal cultures as those used for electrophysiological and immunocytochemical experiments for numerous pre-synaptic proteins by standard Western blotting. Protein expression levels were similar between genotypes by semi-quantitative western blot (relative to GAPDH, Figures 4A,B); by paired *t*-test there were no significant differences between NT littermate and KI cultures in the levels of EndoA (NT = 1.00 ± 0.11, KI = 0.98 ± 0.11, *p* = 0.69), VAMP1 (NT = 1.00 ± 0.22, KI = 0.98 ± 0.19, *p* = 0.90), VAMP2 (NT = 1.00 ± 0.14, KI = 0.90 ± 0.06, *p* = 0.30), synaptojanin 1 (NT = 1.00 ± 0.15 and KI = 0.95 ± 0.16, *p* = 0.72), dynamin 1 (NT = 1.00 ± 0.07 and KI = 1.05 ± 0.14, *p* = 0.37) or synapsin 1 (NT = 1.00 ± 0.31 and KI = 1.31 ± 0.39, *p* = 0.24, Figure 4B).

Synapsins are among the most abundant regulatory synaptic vesicle phosphoproteins, and their function is regulated by kinase and phosphatase activity (Jovanovic et al., 2001; Hojjati et al., 2007; Valente et al., 2012). By standard semi-quantitative western blot, we probed for phosphorylated synapsin 1 (pSyn1) with S9 (site 1) and S603 (site 3) phospho-selective antibodies, and found that the relative levels of phosphorylation at both of these sites was significantly reduced in cortical cultures from KI mice, with respect to NT controls (Figure 4B). Within genotype, Syn1a and Syn1b levels were similar, as were the total and relative phosphorylation levels; in light of this only total Syn1 (a+b) is presented. Reductions in pSyn1, while significant at both sites in KI mice, were derived from standard semi-quantitative western blotting, which suffers from a relatively high degree of variability both between and within sample runs, likely due to minor inconsistencies in liquid handling, sample transfer and human error. In order to confirm reductions in pSyn1 ratios, we ran a subset of KI samples through a *Wes* size-separation (ProteinSimple^©^) assay. This technology uses an automated capillary-based separation process that removes many manual and technical manipulations, including transfer, eliminating much variability and providing direct protein quantification. Clear separation of GAPDH, and Syn1a and b were achieved within each capillary (representative band analysis in Figure 4C) and quantification (Figure 4D) confirmed the results of the standard blotting; Syn1 a and b levels were equivalent, but the ratio of pS603 Syn1 was significantly reduced in KI neurons, relative to NT littermate cultures.

Together the data demonstrate that synaptic activity and protein regulators of vesicle release are altered by the presence of physiological levels of the LRRK2 G2019S mutation in 3-week-old mammalian neurons.

## Discussion

### LRRK2 and regulation of synaptic function *in vitro*

The chronic loss of LRRK2 in KO mouse cortical cultures resulted in only a subtle reduction in glutamatergic transmission at a similar density of synapses at 21 days *in vitro*. Although synaptic cluster densities were unaltered in KO cells, there was a marked general reduction in both VGluT1 and PSD95 signal intensity, which may reflect reduced synaptic protein levels. In light of this, a harsh image threshold would produce a reduction in the density of both markers and synapses in KOs, but careful (blinded) thresholding demonstrated that the size and density of synapses is equivalent in NT and KO cells. KOs cells have been shown to have (at least at some point in development) longer dendrites (MacLeod et al., 2006; Parisiadou et al., 2009; Dachsel et al., 2010; Sepulveda et al., 2013). If they were similarly longer in this study, with equivalent synapse densities, elevations in total synapse number might be predicted to result in an increased event frequency. The opposite trend was observed here in KO cells. Together, the data provide further evidence that LRRK2 acts at glutamatergic synapses in mammalian neurons (Piccoli et al., 2011; Parisiadou et al., 2014) and, along with recent *Drosophila* studies (Lee et al., 2010; Matta et al., 2012), strengthens the case for LRRK2s role in the regulation of synaptic release machinery. That said, understanding the experimental and synaptic context of LRRK2 manipulations is crucial to attempts at extrapolating its physiological role, as both increases (Piccoli et al., 2011) and decreases (Matta et al., 2012; Parisiadou et al., 2014) in synaptic activity have been observed following LRRK2 loss of function in different systems.

Increasing LRRK2 levels 2–3 fold resulted in an increase in the density of synaptic markers and synapse numbers. There were non-significant trends toward increased event frequency and reduced inter-event intervals that may reflect the ~17% increase in synapse density. Alternatively, homeostatic compensation may mask the increased synapse density by reducing probability of release at a greater number of synapses. This is in agreement with a lack of effect observed in DA cell degeneration in *Drosophila* (Lee et al., 2007); however, overexpression has been shown to decrease pre-synaptic bouton numbers (Lee et al., 2010) and have exactly the same effect as LRRK2 loss of function on synaptic release in *Drosophila* (Matta et al., 2012). Together the data strongly suggest that the synaptic consequences of LRRK2 manipulations may be model-, cell- and context-dependent; thus it may be of paramount importance to determine the comparative effects of knock-out, overexpression and mutation within the same system to enable interpretation of the results.

### Physiological levels of G2019S LRRK2 increase glutamate release and alter pre-synaptic function

Examination of overexpression and knock-out of LRRK2 in our primary cortical cultures provided the platform against which to compare the effects of mutant LRRK2. We observed a marked ~37% increase in the frequency of glutamate excitatory currents in G2019S KI cultures, in the absence of any change to synapse density. Importantly, this demonstrated that the LRRK2 mutation produces effects that are distinct from those of simple loss-of function or gain-of-function. We did detect a (non-significant) 13% increase in KI membrane capacitance that could be predictive of increased dendritic membrane area, longer dendrites and more numerous (equally dense) synapses. However, correlation analysis between membrane capacitance and mEPSC event frequency in KI cells showed absolutely no relationship (Pearson's *R* = 0.002, *P* = 0.99), whereas there was a significant positive correlation in NT cells (*R* = 0.49, *P* = 0.007). We take this as evidence that the increase in KI frequency is independent of potential difference in total dendrite length especially as it supersedes the usual correlation—i.e. smaller cells with similar synapse densities also have high event frequency, presumably from overactive pre-synaptic elements. Also, there is consensus from several studies that wild-type overexpression and mutant overexpression both result in shortened dendritic length (MacLeod et al., 2006; Parisiadou et al., 2009; Dachsel et al., 2010; Ramonet et al., 2011; Winner et al., 2011; Sepulveda et al., 2013). If OE and KI cells here also have shortened dendrites, with equal synapse densities, then total synapse number would be reduced. Consequently the observed increase in KI event frequency would be an underestimate for increased Pr.

There was also a significant slowing of membrane responses to direct current injection in KI cells, which correlated with significantly slower mEPSCs rise times. This, in concert with trends in capacitance values, suggests altered intrinsic membrane properties that are likely related and may be explored in future studies. However, like capacitance, there was no significant correlation between mEPSC rise time and event frequency in either NT (*R* = 0.1, *p* = 0.09) or KI cells (*R* = 0.004, *p* = 0.7), arguing that post-synaptic membrane alterations do not account for increased transmission. It appears that a single copy of G2019S LRRK2 is sufficient to dramatically alter excitatory synaptic release, in a manner distinct from a loss of LRRK2 and in excess of any changes produced by a 3-fold increase in LRRK2 levels.

The G2019S mutation resides in LRRK2's kinase domain, and has been shown to augment LRRK2 kinase activity *in vitro*, demonstrating a gain-of-function for LRRK2 autophosporylation and phosphorylation of a generic substrate (West et al., 2005; Nichols et al., 2010). This has led to a major push for identification of LRRK2 substrates and the development of kinase inhibitors as they may offer therapeutic potential (Webber et al., 2011). The list of candidate substrates is growing, and includes Tau (Kawakami et al., 2012; Bailey et al., 2013), 4E-BP (Lee et al., 2010), and EndoA (Matta et al., 2012). Unfortunately, interpretation of many of these findings is hampered by binding relationships potentially forced *in vitro* by non-physiological concentrations of substrate and enzyme (Webber et al., 2011), failure of supporting evidence *in vivo* (Trancikova et al., 2012) and reliance on inhibitors that exhibit off-target and/or systemic effects (Drolet et al., 2011; Cirnaru et al., 2014; Luerman et al., 2014) even in LRRK2 knock-out cells. That said, it is clear that many of the proposed LRRK2 interactors and substrates are directly linked to the synaptic vesicle cycle, notably syntaxin 1A, dynamin1, synapsin1 and VAMP2 (Piccoli et al., 2014) and EndoA; phosphorylation of EndoA by LRRK2 has been demonstrated to regulate transmitter release (Matta et al., 2012). If the cause of increased release is as simple as the ~3-fold increase in LRRK2 kinase activity (West et al., 2005; Nichols et al., 2010), other factors must be at play to account for synaptic alterations in KI mouse cells well above those seen in OE cultures (expressing 3-fold more LRRK2) and preferential effects upon glutamatergic, rather than GABAergic release. LRRK2 localization and kinase activity are regulated by its own phosphorylation state and through dimerization by co-chaperone 14-3-3 (Sen et al., 2009; Nichols et al., 2010; Rudenko and Cookson, 2010); thus greater effects may be engendered by G2019S upon kinase activity in living neuronal systems under appropriate regulation. We assayed the protein levels of several interactors but found none to be significantly altered. The phosphorylation state of vesicle cycle regulators has direct consequences for their activity and we sought to assay the phosphorylation status of EndoA, pertinent to LRRK2 activity and vesicle release in *Drosophila* (Matta et al., 2012); unfortunately, the only phosphoantibody currently specific to the pertinent EndoA serine 75 site is ineffective in mammalian tissue (personal communication, Dr. Patrik Verstreken). We therefore turned our attention to reasonably well-characterized phosphorylation sites on synapsin 1, one of the most abundant of all pre-synaptic vesicle proteins.

Synapsins are believed to regulate the balance between the reserve and the readily releasable (docked) vesicle pools and act as modulators of vesicle exocytosis (Fdez and Hilfiker, 2006). It has been suggested that phosphorylated synapsin 1 binds vesicles and tethers them to the actin cytoskeleton for retention in the reserve pool; thus, phospho-synapsin 1 reduces the number of vesicles available for release from the readily releasable pool, whereas dephosphorylated synapsin dissociates from vesicles, thereby freeing them to dock for ready release (Hosaka et al., 1999). However, this supposition is not universally supported; some reports describe synapsin depletion when vesicles undergo active zone docking (Pieribone et al., 1995) but others show that synapsins remain associated with vesicles during exo- and endocytosis (Torri-Tarelli et al., 1990). Furthermore, with several studies showing the mature vesicle cluster contains virtually no cytoskeleton, the original hypothesis is unlikely to explain synapsin function there (reviewed in Sudhof, 2004; Fdez and Hilfiker, 2006). Although the mechanism by which synapsin 1 regulates vesicle release remains elusive, synapsin 1 phosphorylation states are indicative of pre-synaptic regulation and release activity (reviewed in Sudhof, 2004; Fdez and Hilfiker, 2006; Valente et al., 2012; Verstegen et al., 2014). In KI cortical cultures that exhibited a marked increase in synaptic release, we found a clear reduction in synapsin 1 phosphorylation at both S6 and S603 by traditional western blot and confirmed reduced pS603 by proteinsimple *Wes* size separation.

It is interesting to note that we observed significant effects upon glutamate frequency and significant effects upon GABA amplitudes. An increase in excitatory synaptic transmission across the neuronal network in the culture might be predicted to alter the GABAergic inhibitory neurons within it and subsequently the post-synaptic responsiveness to their activity (Wang and Maffei, 2014). Homeostatic mechanisms may also exaggerate GABAergic inhibition, in order to counteract the effects of increased glutamate release (Shepherd et al., 2006; Maffei and Fontanini, 2009), but network interactions are very hard to predict or interpret. This dichotomy may even be explained by alterations to synapsin 1, as it has been shown that neurons in cortical cultures prepared from synapsin 1 null mice exhibit opposite changes to glutamate and GABA transmission (Chiappalone et al., 2009).

The S603 site is a specific target of CaMKII and, predictably, synapsin 1 pS603 is reduced by ~70% in CaMKII knock-out mice (Hojjati et al., 2007). In support of our link between reduced pS603 and increased release, CaMKII knock-out mice have an increased probability of release (Silva et al., 1992; Hinds et al., 2003), significantly more docked vesicles and a reduced propensity to synaptic fatigue (Hojjati et al., 2007). Probing the similarity between LRRK2 G2019S KI mice and CaMKII knock-outs may prove interesting in future studies.

### Summary and conclusions

Together, the evidence collected here in cortical cultures from KO, OE and KI mice demonstrates that LRRK2 has an influence on pre-synaptic function, likely through regulation of pre-synaptic regulatory proteins. The challenge is to clarify which specific functions of LRRK2 go awry in G2019S mutants, and how this eventually leads to parkinsonism. The data compliment and extend the contemporary literature (Piccoli et al., 2011; Matta et al., 2012; Parisiadou et al., 2014) by providing evidence in support of a role for LRRK2 in synaptic transmission and a clear gain-of-function effect of the G2019S mutation. Furthermore, the increases in synaptic release in G2019S KI mice are distinct from, or in excess of, those produced by a 3-fold increase in LRRK2 protein levels (in OE mice).

Our data also show that the loss of LRRK2 is tolerated in 3-week-old neuronal cultures and the brain, as predicted from viable, generally healthy LRRK2 KO mice (Hinkle et al., 2012). Therefore, it seems likely that LRRK2 exhibits a high degree of functional redundancy in the central nervous system (CNS) and that loss-of-function effects are unlikely to underlie PD pathogenesis. In light of this, CNS-specific silencing of LRRK2 may present a viable therapeutic target.

### Conflict of interest statement

The authors declare that the research was conducted in the absence of any commercial or financial relationships that could be construed as a potential conflict of interest.

## References

[B1] Bailey R. M. Covy J. P. Melrose H. L. Rousseau L. Watkinson R. Knight J. (2013). LRRK2 phosphorylates novel tau epitopes and promotes tauopathy. Acta Neuropathol. 126, 809–827 10.1007/s00401-013-1188-4 24113872 PMC3830748

[B2] Biskup S. Moore D. J. Rea A. Lorenz-Deperieux B. Coombes C. E. Dawson V. L. (2007). Dynamic and redundant regulation of LRRK2 and LRRK1 expression. BMC Neurosci. 8:102 10.1186/1471-2202-8-102 18045479 PMC2233633

[B3] Brigidi G. S. Sun Y. Beccano-Kelly D. Pitman K. Mobasser M. Borgland S. L. (2014). Palmitoylation of delta-catenin by DHHC5 mediates activity-induced synapse plasticity. Nat. Neurosci. 17, 522–532 10.1038/nn.3657 24562000 PMC5025286

[B4] Chan D. Citro A. Cordy J. M. Shen G. C. Wolozin B. (2011). Rac1 protein rescues neurite retraction caused by G2019S leucine-rich repeat kinase 2 (LRRK2). J. Biol. Chem. 286, 16140–16149 10.1074/jbc.M111.234005 21454543 PMC3091223

[B5] Chiappalone M. Casagrande S. Tedesco M. Valtorta F. Baldelli P. Martinoia S. (2009). Opposite changes in glutamatergic and GABAergic transmission underlie the diffuse hyperexcitability of synapsin I-deficient cortical networks. Cereb. Cortex 19, 1422–1439 10.1093/cercor/bhn182 19020204

[B6] Cirnaru M. D. Marte A. Belluzzi E. Russo I. Gabrielli M. Longo F. (2014). LRRK2 kinase activity regulates synaptic vesicle trafficking and neurotransmitter release through modulation of LRRK2 macro-molecular complex. Front. Mol. Neurosci. 7:49 10.3389/fnmol.2014.00049 24904275 PMC4034499

[B7] Cookson M. R. (2010). The role of leucine-rich repeat kinase 2 (LRRK2) in Parkinson's disease. Nature Rev. 11, 791–797 10.1038/nrn2935 21088684 PMC4662256

[B8] Dachsel J. C. Behrouz B. Yue M. Beevers J. E. Melrose H. L. Farrer M. J. (2010). A comparative study of Lrrk2 function in primary neuronal cultures. Parkinsonism Relat. Disord. 16, 650–655 10.1016/j.parkreldis.2010.08.018 20850369 PMC3159957

[B9] Drolet R. E. Sanders J. M. Kern J. T. (2011). Leucine-rich repeat kinase 2 (LRRK2) cellular biology: a review of recent advances in identifying physiological substrates and cellular functions. J. Neurogenet. 25, 140–151 10.3109/01677063.2011.627072 22077787

[B10] Fdez E. Hilfiker S. (2006). Vesicle pools and synapsins: new insights into old enigmas. Brain Cell Biol. 35, 107–115 10.1007/s11068-007-9013-4 17957477

[B11] Herzig M. C. Kolly C. Persohn E. Theil D. Schweizer T. Hafner T. (2011). LRRK2 protein levels are determined by kinase function and are crucial for kidney and lung homeostasis in mice. Hum. Mol. Genet. 20, 4209–4223 10.1093/hmg/ddr348 21828077 PMC3188995

[B12] Hinds H. L. Goussakov I. Nakazawa K. Tonegawa S. Bolshakov V. Y. (2003). Essential function of alpha-calcium/calmodulin-dependent protein kinase II in neurotransmitter release at a glutamatergic central synapse. Proc. Natl. Acad. Sci. U.S.A. 100, 4275–4280 10.1073/pnas.0530202100 12629219 PMC153083

[B13] Hinkle K. M. Yue M. Behrouz B. Dachsel J. C. Lincoln S. J. Bowles E. E. (2012). LRRK2 knockout mice have an intact dopaminergic system but display alterations in exploratory and motor co-ordination behaviors. Mol. Neurodegener. 7:25 10.1186/1750-1326-7-25 22647713 PMC3441373

[B14] Hojjati M. R. van Woerden G. M. Tyler W. J. Giese K. P. Silva A. J. Pozzo-Miller L. (2007). Kinase activity is not required for alphaCaMKII-dependent presynaptic plasticity at CA3-CA1 synapses. Nat. Neurosci. 10, 1125–1127 10.1038/nn1946 17660813 PMC2804046

[B15] Hosaka M. Hammer R. E. Sudhof T. C. (1999). A phospho-switch controls the dynamic association of synapsins with synaptic vesicles. Neuron 24, 377–387 10.1016/S0896-6273(00)80851-X 10571231

[B16] Jovanovic J. N. Sihra T. S. Nairn A. C. Hemmings H. C. Jr. Greengard P. Czernik A. J. (2001). Opposing changes in phosphorylation of specific sites in synapsin I during Ca2+-dependent glutamate release in isolated nerve terminals. J. Neurosci. 21, 7944–7953 11588168 10.1523/JNEUROSCI.21-20-07944.2001 PMC6763853

[B17] Kaufman A. M. Milnerwood A. J. Sepers M. D. Coquinco A. She K. Wang L. (2012). Opposing roles of synaptic and extrasynaptic NMDA receptor signaling in cocultured striatal and cortical neurons. J. Neurosci. 32, 3992–4003 10.1523/JNEUROSCI.4129-11.2012 22442066 PMC6621208

[B18] Kawakami F. Yabata T. Ohta E. Maekawa T. Shimada N. Suzuki M. (2012). LRRK2 phosphorylates tubulin-associated tau but not the free molecule: LRRK2-mediated regulation of the tau-tubulin association and neurite outgrowth. PLoS ONE 7:e30834 10.1371/journal.pone.0030834 22303461 PMC3267742

[B19] Lee S. B. Kim W. Lee S. Chung J. (2007). Loss of LRRK2/PARK8 induces degeneration of dopaminergic neurons in Drosophila. Biochem. Biophys. Res. Commun. 358, 534–539 10.1016/j.bbrc.2007.04.156 17498648

[B20] Lee S. Imai Y. Gehrke S. Liu S. Lu B. (2012). The synaptic function of LRRK2. Biochem. Soc. Trans. 40, 1047–1051 10.1042/BST20120113 22988863

[B21] Lee S. Liu H. P. Lin W. Y. Guo H. Lu B. (2010). LRRK2 kinase regulates synaptic morphology through distinct substrates at the presynaptic and postsynaptic compartments of the Drosophila neuromuscular junction. J. Neurosci. 30, 16959–16969 10.1523/JNEUROSCI.1807-10.2010 21159966 PMC3045823

[B22] Lin C. H. Tsai P. I. Wu R. M. Chien C. T. (2010). LRRK2 G2019S mutation induces dendrite degeneration through mislocalization and phosphorylation of tau by recruiting autoactivated GSK3ss. J. Neurosci. 30, 13138–13149 10.1523/JNEUROSCI.1737-10.2010 20881132 PMC6633523

[B23] Luerman G. C. Nguyen C. Samaroo H. Loos P. Xi H. Hurtado-Lorenzo A. (2014). Phosphoproteomic evaluation of pharmacological inhibition of leucine-rich repeat kinase 2 reveals significant off-target effects of LRRK-2-IN-1. J. Neurochem. 128, 561–576 10.1111/jnc.12483 24117733

[B24] MacLeod D. Dowman J. Hammond R. Leete T. Inoue K. Abeliovich A. (2006). The familial Parkinsonism gene LRRK2 regulates neurite process morphology. Neuron 52, 587–593 10.1016/j.neuron.2006.10.008 17114044

[B25] Maffei A. Fontanini A. (2009). Network homeostasis: a matter of coordination. Curr. Opin. Neurobiol. 19, 168–173 10.1016/j.conb.2009.05.012 19540746 PMC3427905

[B26] Matta S. Van Kolen K. da Cunha R. van den Bogaart G. Mandemakers W. Miskiewicz K. (2012). LRRK2 controls an EndoA phosphorylation cycle in synaptic endocytosis. Neuron 75, 1008–1021 10.1016/j.neuron.2012.08.022 22998870

[B27] Melrose H. L. Dachsel J. C. Behrouz B. Lincoln S. J. Yue M. Hinkle K. M. (2010). Impaired dopaminergic neurotransmission and microtubule-associated protein tau alterations in human LRRK2 transgenic mice. Neurobiol. Dis. 40, 503–517 10.1016/j.nbd.2010.07.010 20659558 PMC2955774

[B28] Milnerwood A. J. Kaufman A. M. Sepers M. D. Gladding C. M. Zhang L. Wang L. (2012). Mitigation of augmented extrasynaptic NMDAR signaling and apoptosis in cortico-striatal co-cultures from Huntington's disease mice. Neurobiol. Dis. 48, 40–51 10.1016/j.nbd.2012.05.013 22668780

[B29] Mizuno-Yamasaki E. Rivera-Molina F. Novick P. (2012). GTPase networks in membrane traffic. Annu. Rev. Biochem. 81, 637–659 10.1146/annurev-biochem-052810-093700 22463690 PMC3708692

[B30] Nichols R. J. Dzamko N. Morrice N. A. Campbell D. G. Deak M. Ordureau A. (2010). 14-3-3 binding to LRRK2 is disrupted by multiple Parkinson's disease-associated mutations and regulates cytoplasmic localization. Biochem. J. 430, 393–404 10.1042/BJ20100483 20642453 PMC2932554

[B31] O'Neill R. A. Bhamidipati A. Bi X. Deb-Basu D. Cahill L. Ferrante J. (2006). Isoelectric focusing technology quantifies protein signaling in 25 cells. Proc. Natl. Acad. Sci. U.S.A. 103, 16153–16158 10.1073/pnas.0607973103 17053065 PMC1618307

[B32] Parisiadou L. Xie C. Cho H. J. Lin X. Gu X. L. Long C. X. (2009). Phosphorylation of ezrin/radixin/moesin proteins by LRRK2 promotes the rearrangement of actin cytoskeleton in neuronal morphogenesis. J. Neurosci. 29, 13971–13980 10.1523/JNEUROSCI.3799-09.2009 19890007 PMC2807632

[B33] Parisiadou L. Yu J. Sgobio C. Xie C. Liu G. Sun L. (2014). LRRK2 regulates synaptogenesis and dopamine receptor activation through modulation of PKA activity. Nat. Neurosci. 17, 367–376 10.1038/nn.3636 24464040 PMC3989289

[B34] Piccoli G. Condliffe S. B. Bauer M. Giesert F. Boldt K. De Astis S. (2011). LRRK2 controls synaptic vesicle storage and mobilization within the recycling pool. J. Neurosci. 31, 2225–2237 10.1523/JNEUROSCI.3730-10.2011 21307259 PMC6633036

[B35] Piccoli G. Onofri F. Cirnaru M. D. Kaiser C. J. Jagtap P. Kastenmuller A. (2014). Leucine-rich repeat kinase 2 binds to neuronal vesicles through protein interactions mediated by its C-terminal WD40 domain. Mol. Cell. Biol. 34, 2147–2161 10.1128/MCB.00914-13 24687852 PMC4054300

[B36] Pieribone V. A. Shupliakov O. Brodin L. Hilfiker-Rothenfluh S. Czernik A. J. Greengard P. (1995). Distinct pools of synaptic vesicles in neurotransmitter release. Nature 375, 493–497 10.1038/375493a0 7777058

[B37] Plowey E. D. Cherra S. J. 3rd. Liu Y. J. Chu C. T. (2008). Role of autophagy in G2019S-LRRK2-associated neurite shortening in differentiated SH-SY5Y cells. J. Neurochem. 105, 1048–1056 10.1111/j.1471-4159.2008.05217.x 18182054 PMC2361385

[B38] Ramonet D. Daher J. P. Lin B. M. Stafa K. Kim J. Banerjee R. (2011). Dopaminergic neuronal loss, reduced neurite complexity and autophagic abnormalities in transgenic mice expressing G2019S mutant LRRK2. PLoS ONE 6:e18568 10.1371/journal.pone.0018568 21494637 PMC3071839

[B39] Rudenko I. N. Cookson M. R. (2010). 14-3-3 proteins are promising LRRK2 interactors. Biochem. J. 430, e5–e6 10.1042/BJ20101200 20795948

[B40] Sen S. Webber P. J. West A. B. (2009). Dependence of leucine-rich repeat kinase 2 (LRRK2) kinase activity on dimerization. J. Biol. Chem. 284, 36346–36356 10.1074/jbc.M109.025437 19826009 PMC2794750

[B41] Sepulveda B. Mesias R. Li X. Yue Z. Benson D. L. (2013). Short- and long-term effects of LRRK2 on axon and dendrite growth. PLoS ONE 8:e61986 10.1371/journal.pone.0061986 23646112 PMC3640004

[B42] Shepherd J. D. Rumbaugh G. Wu J. Chowdhury S. Plath N. Kuhl D. (2006). Arc/Arg3.1 mediates homeostatic synaptic scaling of AMPA receptors. Neuron 52, 475–484 10.1016/j.neuron.2006.08.034 17088213 PMC1764219

[B43] Silva A. J. Stevens C. F. Tonegawa S. Wang Y. (1992). Deficient hippocampal long-term potentiation in alpha-calcium-calmodulin kinase II mutant mice. Science 257, 201–206 10.1126/science.1378648 1378648

[B44] Stafa K. Tsika E. Moser R. Musso A. Glauser L. Jones A. (2014). Functional interaction of Parkinson's disease-associated LRRK2 with members of the dynamin GTPase superfamily. Hum. Mol. Genet. 23, 2055–2077 10.1093/hmg/ddt600 24282027 PMC3959816

[B45] Sudhof T. C. (2004). The synaptic vesicle cycle. Annu. Rev. Neurosci. 27, 509–547 10.1146/annurev.neuro.26.041002.131412 15217342

[B46] Tapia L. Milnerwood A. Guo A. Mills F. Yoshida E. Vasuta C. (2011). Progranulin deficiency decreases gross neural connectivity but enhances transmission at individual synapses. J. Neurosci. 31, 11126–11132 10.1523/JNEUROSCI.6244-10.2011 21813674 PMC6623368

[B47] Torri-Tarelli F. Villa A. Valtorta F. De Camilli P. Greengard P. Ceccarelli B. (1990). Redistribution of synaptophysin and synapsin I during alpha-latrotoxin-induced release of neurotransmitter at the neuromuscular junction. J. Cell Biol. 110, 449–459 10.1083/jcb.110.2.449 1967610 PMC2116013

[B48] Trancikova A. Mamais A. Webber P. J. Stafa K. Tsika E. Glauser L. (2012). Phosphorylation of 4E-BP1 in the mammalian brain is not altered by LRRK2 expression or pathogenic mutations. PLoS ONE 7:e47784 10.1371/journal.pone.0047784 23082216 PMC3474772

[B49] Valente P. Casagrande S. Nieus T. Verstegen A. M. Valtorta F. Benfenati F. (2012). Site-specific synapsin I phosphorylation participates in the expression of post-tetanic potentiation and its enhancement by BDNF. J. Neurosci. 32, 5868–5879 10.1523/JNEUROSCI.5275-11.2012 22539848 PMC6703602

[B50] Verstegen A. M. Tagliatti E. Lignani G. Marte A. Stolero T. Atias M. (2014). Phosphorylation of synapsin I by cyclin-dependent kinase-5 sets the ratio between the resting and recycling pools of synaptic vesicles at hippocampal synapses. J. Neurosci. 34, 7266–7280 10.1523/JNEUROSCI.3973-13.2014 24849359 PMC6608192

[B51] Wang L. Maffei A. (2014). Inhibitory plasticity dictates the sign of plasticity at excitatory synapses. J. Neurosci. 34, 1083–1093 10.1523/JNEUROSCI.4711-13.2014 24453301 PMC3898280

[B52] Webber P. J. Smith A. D. Sen S. Renfrow M. B. Mobley J. A. West A. B. (2011). Autophosphorylation in the leucine-rich repeat kinase 2 (LRRK2) GTPase domain modifies kinase and GTP-binding activities. J. Mol. Biol. 412, 94–110 10.1016/j.jmb.2011.07.033 21806997 PMC3158845

[B53] West A. B. Moore D. J. Biskup S. Bugayenko A. Smith W. W. Ross C. A. (2005). Parkinson's disease-associated mutations in leucine-rich repeat kinase 2 augment kinase activity. Proc. Natl. Acad. Sci. U.S.A. 102, 16842–16847 10.1073/pnas.0507360102 16269541 PMC1283829

[B54] Winner B. Melrose H. L. Zhao C. Hinkle K. M. Yue M. Kent C. (2011). Adult neurogenesis and neurite outgrowth are impaired in LRRK2 G2019S mice. Neurobiol. Dis. 41, 706–716 10.1016/j.nbd.2010.12.008 21168496 PMC3059106

